# Adequacy of public health communications on H7N9 and MERS in Singapore: insights from a community based cross-sectional study

**DOI:** 10.1186/s12889-018-5340-x

**Published:** 2018-04-02

**Authors:** Yan’an Hou, Yi-roe Tan, Wei Yen Lim, Vernon Lee, Linda Wei Lin Tan, Mark I-Cheng Chen, Peiling Yap

**Affiliations:** 1 Saw Swee Hock School of Public Health, National University of Singapore, Tahir Foundation Building, 12 Science Drive 2, #10-01, Singapore, 117549 Singapore; 2 Epidemiology Unit, Division of Infectious Diseases, Department of Medicine, National University Hospital Singapore, Singapore, Singapore; 3 Institute of Infectious Diseases and Epidemiology, Tan Tock Seng Hospital, Singapore, Singapore; 4 Research and Development Office, Agency for Integrated Care, Singapore, Singapore

**Keywords:** Outbreaks, Emerging infections, Public communication, H7N9, MERS

## Abstract

**Background:**

Singapore remains vulnerable to worldwide epidemics due to high air traffic with other countries This study aims to measure the public’s awareness of the Middle East Respiratory Syndrome (MERS) and Avian Influenza A (H7N9), identify population groups who are uninformed or misinformed about the diseases, understand their choice of outbreak information source, and assess the effectiveness of communication channels in Singapore.

**Methods:**

A cross-sectional study, comprising of face-to-face interviews, was conducted between June and December 2013 to assess the public’s awareness and knowledge of MERS and H7N9, including their choice of information source. Respondents were randomly selected and recruited from 3 existing cohort studies. An opportunistic sampling approach was also used to recruit new participants or members in the same household through referrals from existing participants.

**Results:**

Out of 2969 participants, 53.2% and 79.4% were not aware of H7N9 and MERS respectively. Participants who were older and better educated were most likely to hear about the diseases. The mean total knowledge score was 9.2 (S.D ± 2.3) out of 20, and 5.9 (S.D ± 1.2) out of 10 for H7N9 and MERS respectively. Participants who were Chinese, more educated and older had better knowledge of the diseases. Television and radio were the primary sources of outbreak information regardless of socio-demographic factors.

**Conclusion:**

Heightening education of infectious outbreaks through appropriate media to the young and less educated could increase awareness.

## Background

Following the global influenza pandemic caused by the novel influenza A virus in 2009 (H1N1–2009), the world continues to be threatened by emerging respiratory diseases such as the Middle East Respiratory Syndrome (MERS) in 2012 and Avian Influenza A (H7N9) in 2013 [1, 2]. The onset of MERS and H7N9 infections in humans are typically characterized by high fever (≥ 38 °C) and cough, and can lead to progressive pneumonia, respiratory failure and death, contributing to case fatality rates of 35% and 19% respectively at the time when this study was performed [2–4].

Such infectious diseases are of concern due to global travel and high volumes of air traffic. Since the first reported case in China, imported cases of H7N9 have been observed in Hong Kong, Taiwan, Canada and Malaysia [5–7]. While human-to-human transmission is limited [8], Singapore remains vulnerable to imported cases of H7N9 due to high air traffic between China and Singapore. In the first half of 2014, Singapore received close to a million tourists from China [9], while China ranks as the third most frequent outbound travel destination among Singaporeans [10]. Similarly, despite the fact that most MERS cases have been reported in the Arabian Peninsula and sustained community transmission has not been documented, imported cases have occurred in the region including in Malaysia, Thailand and the Republic of Korea [11]. Although both H7N9 and MERS were not epidemic at the time of the study and knowledge available on both diseases were limited to global news and reports, given Singapore’s position as an international trading and traveling hub, it is still important to convey to the public accurate and timely information on the nature of the infectious outbreak, its transmission modes and preventive measures so as to better prepare them for potential epidemics.

The International Health Regulations (2005) states risk communication as one of the eight core capacities for outbreak preparedness [12]. For the planning of effective risk communication, it is necessary to assess the public’s knowledge level to determine vulnerable target groups. Multiple cross-sectional studies have been done to assess knowledge and attitude of the public on past respiratory disease outbreaks [13–18]. Ethnicity, age and education level were found to influence an individual’s knowledge level of infectious outbreaks. These studies strongly indicate the need to consider audience segmentation along with appropriate use of media channels, allowing for tailored public health messages to be delivered timely and accurately [19, 20].

Understanding how the public gathers information on infectious diseases, and what media channels are preferred to deliver customized messages before an outbreak, equips the government with useful information for risk communication planning [21, 22]. Credible and timely message delivery through appropriate media channels is necessary to ensure the public gets accurate information on emerging infectious diseases to make informed decisions on protective health behaviors [23, 24]. Studies have also shown that inconsistent and untargeted risk communication messages may result in gaps in health-related knowledge and eventually, health outcomes [25–27].

The objective of this study is to identify the groups most likely to be uninformed or misinformed about H7N9 and MERS infection, as well as to determine appropriate media channels for public health education in Singapore. It also aims to assess if the public are given sufficient information to take specific preventive measures to protect themselves. Findings from the study will help health promotion agencies develop effective communication strategies to mitigate the risk of future emerging infectious agents.

## Methods

### Sample

A cross-sectional community-based survey was carried out in Singapore, a densely-populated (7987 people/km^2^) tropical island city-state with a total population of 5.61 million [28], from June to December 2013. The participants were recruited from 3 existing studies: 1) Singapore Health 2012 [29], 2) Saw Swee Hock School of Public Health (SSHSPH) Revisit of the Multi-ethnic and Diabetic Cohorts [30] and 3) Revisiting the Singapore Consortium of Cohort Studies – Multi-ethnic cohort. Respondents were randomly selected and contacted via phone to explain the nature of the study and invited to participate. In addition, an opportunistic sampling approach was also used to recruit new participants or members in the same household through referrals from existing participants.

Although the main aim of the study was to determine the adequacy of public health communications on H7N9 and MERS in Singapore, it was also planned that in the event of a community outbreak, the study population will allow us to establish the attack rate of either virus through a sero-epidemiological investigation. Hence, assuming a true proportion of 10% and at 95% confidence, a sample size of 3000 (500 participants under 21 years, 2000 participants between 21 and 55 years and 500 participants above 55 years) was calculated to allow for sufficient power to estimate the proportion of population infected with either disease to a precision of 2.6% for the young and old and 1.3% for those aged between 21 and 55 years. The calculated sample size was also deemed sufficient for the questionnaire.

### Instrument

The paper-based questionnaire was adapted from a literature review of published articles on knowledge of H1N1 and H5N1 [31–33], as well as existing questions from the Chinese Centre for Disease Control and Prevention. The Health Belief Model was also employed so as to include questions on perceived susceptibility, severity and benefits and cues to action [34]. A small pilot test was conducted among staff of the SSHSPH to fine-tune the questionnaire such that it is adapted to the local culture and language. Demographic questions relating to gender, age group, ethnicity, housing type (as more than 80% of the local residents live in public housing [35], further stratification between public and private housing could provide insights to the socio-economic status of our study population) and education level were asked before the survey started. A 15–20 min face-to-face survey was conducted by a team of trained multi-ethnic interviewers at the participant’s home or a place of their choice. The questionnaires were conducted in one of the four official languages of Singapore, based on the participant’s preference: English, Mandarin, Malay and Tamil.

Participants were asked if they have ever heard of H7N9 and/or MERS and their preferred information source about infectious disease outbreaks, with traditional media channels, social media and word of mouth from family, friends and colleagues as possible options. Participants could choose more than one preferred information source. For the knowledge assessment of H7N9, participants were asked to answer ‘yes’, ‘no’ or ‘not sure’ on two sections, namely the scientific understanding and transmission modes of H7N9, which also include methods to reduce risk of seasonal flu and being infected with H7N9 in particular. For the knowledge assessment of MERS, participants were also asked to answer ‘yes’, ‘no’ or ‘not sure’ on two sections, namely scientific understanding and transmission modes of MERS.

### Data analysis

Data were double-entered and cross-checked using Excel version 2013 (Microsoft Corp.; Redmond, USA). Statistical analyses were performed using STATA 13.0 (STATA Corp.; College Station, USA). All baseline socio-demographics were described as categorical variables (gender, age group, ethnicity, housing type and education level). Private housing includes condominium/landed/others, while primary education refers to no formal/primary education; secondary education refers to secondary/‘O’/‘A’ level; tertiary education refers to vocational/university and above. A chi-square test was used to determine if there was a statistical difference between participants who had versus those who had never heard of H7N9 and MERS. Multivariable logistic analysis, with Odds Ratios (OR) reported, were used to determine factors associated with awareness of H7N9 and MERS.

The scoring for knowledge of H7N9 and MERS encompassed the participant’s scientific understanding of the diseases, transmission modes and methods of reducing risk of infection. A negative-marking scoring method was used to reflect the participant’s true understanding of the diseases. Correct answers were scored with a positive value of one, incorrect answers were given a negative value of one and questions that were answered ‘not sure’ or omitted were given a value of zero. By proportion, the knowledge scores for H7N9 were scaled to a maximum score of 20 as there were a total of 12 questions and for MERS to a maximum score of 10 as there were a total of 6 questions. Multivariable linear regression analysis was used to determine factors associated with the summative H7N9 and MERS knowledge scores among respondents who had heard of H7N9 and/or MERS. Additionally, the study also analyzed key questions relating to the transmission of H7N9 and MERS to understand how well the participants knew about specific preventive measures to protect themselves; descriptive statistics was used for this with results expressed as percentages.

As the study population was partially recruited through opportunistic sampling of members in the same households, all logistic and linear regression analysis used a multilevel mixed-effects model with a random intercept to adjust for effects observed due to potential household clustering. Statistical significance was considered at *P* < 0.05 for all analyses.

## Results

### Participant’s characteristics

The socio-demographic characteristics of the 2969 respondents in the survey are described in Table 1. “Table 1 about here” A higher proportion of females was observed in our study population, and the mean age was 42.4 years (range: 16–96 years). Majority of respondents in the sample were of Chinese ethnicity (38.7%) and between the ages of 40–59 years old (38.2%). Majority of the respondents resided in public housing type of 4 rooms and above and about half surveyed had attained at least secondary education.

**Table 1:** Baseline demographics of respondents, stratified into ever and never heard of H7N9 or MERS

*N* = 2969			Ever heard of H7N9 (*N* = 1389, 46.8%)		Ever heard of MERS (*N* = 613, 20.6%)	
Socio-demographic characteristics		Total respondents (%) ^a^	No. of respondents (%) ^b^	*P*-value	No. of respondents (%) ^b^	*P*-value
Gender	Male	1170 (39.4)	568 (48.5)	0.120	259 (22.1)	0.106
	Female	1799 (60.6)	821 (45.6)		354 (19.7)	
Age group	16–21	362 (12.2)	129 (35.6)	< 0.001*	36 (9.9)	< 0.001*
	22–39	962 (32.4)	408 (42.4)		176 (18.3)	
	40–59	1133 (38.2)	601(53.0)		275 (24.3)	
	≥ 60	512 (17.2)	251 (49.0)		126 (24.6)	
Ethnicity	Chinese	1148 (38.7)	676 (58.9)	< 0.001*	243 (21.2)	0.010*
	Malay	1004 (33.8)	376 (37.5)		188 (18.7)	
	Indian	745 (25.1)	298 (40.0)		157 (21.1)	
	Others	72 (2.4)	39 (54.2)		25 (34.7)	
Housing	Public – 3 rooms and below	1043 (35.1)	408 (39.1)	< 0.001*	167 (16.0)	< 0.001*
	Public – 4 rooms and above	1731 (58.3)	875 (50.5)		385 (22.2)	
	Private	195 (6.6)	106 (54.4)		61 (31.3)	
Highest education attainment	Primary	577 (19.4)	210 (36.4)	< 0.001*	68 (11.8)	< 0.001*
	Secondary	1509 (50.8)	693 (45.9)		298 (19.7)	
	Tertiary	883 (29.8)	486 (55.0)		247 (28.0)	

*Significant difference between variable and awareness of H7N9 or MERS at *P* < 0.05, chi-squared test

^a^Tabulated in column percentages

^b^Tabulated in row percentages

### General awareness of H7N9/MERS

As illustrated in Table 1, a larger portion of respondents had never heard of MERS (79.4%) before as compared to H7N9 (53.2%). In terms of age group, 64.4% and 90.1% of the respondents aged 16–21 years had never heard of H7N9 and MERS respectively (*P* < 0.001). Among the different ethnic groups, 41.1% and 78.8% of the Chinese respondents had never heard of H7N9 and MERS respectively (*P* < 0.01). Among respondents living in public housing type of 3 rooms and below, 60.9% and 84.0% of them had never heard of H7N9 and MERS respectively (*P* < 0.001). With regards to education, 63.6% and 88.2% of the respondents with primary education had never heard of H7N9 and MERS respectively (*P* < 0.001). General awareness of both the diseases were not significantly different for both genders.

Multi-level multivariable logistic regression analysis (Table 2) “Table 2 about here” was done to determine factors associated with awareness of H7N9 or MERS. Regarding H7N9, individuals who were 40 years old and above (OR = 3.24, 95% C.I 2.21–4.77) or with at least secondary education (OR = 1.72, 95% C.I 1.25–2.37) were significantly more likely to hear of the disease compared to the reference groups of those aged 16–21 years or with primary education respectively. Compared with the Chinese, the Malay and Indian ethnic groups were significantly less likely to hear about H7N9 (OR = 0.40, 95% C.I 0.29–0.54 and OR = 0.35, 95% C.I 0.25–0.49 respectively). Respondents who relied on printed media or websites/ Internet for outbreak information were more likely (OR = 1.62, 95% C.I 1.23–2.13, and OR = 1.57, 95% C.I 1.16–2.11 respectively), while those who relied on word of mouth from their family members and/or relatives were less likely (OR = 0.58, 95% C.I 0.41–0.82) to have heard of H7N9.

**Table 2:** Multi-level multivariable logistic regression of factors associated with awareness of H7N9 or MERS

	H7N9			MERS		
Variable	Odds ratio	95% CI	*P*-value	Odds ratio	95% CI	*P*-value
Gender						
Male	1.00	Reference		1.00	Reference	
Female	0.86	(0.70, 1.07)	0.169	0.90	(0.72, 1.14)	0.391
Age group						
16–21	1.00	Reference		1.00	Reference	
22–39	1.34	(0.91, 1.97)	0.133	1.76	(1.10, 2.83)	0.019*
40–59	3.24	(2.21, 4.77)	< 0.001*	4.35	(2.71, 6.98)	< 0.001*
≥ 60	2.79	(1.74, 4.49)	< 0.001*	6.00	(3.44, 10.45)	< 0.001*
Ethnicity						
Chinese	1.00	Reference		1.00	Reference	
Malay	0.40	(0.29, 0.54)	< 0.001*	1.48	(1.08, 2.05)	0.016*
Indian	0.35	(0.25, 0.49)	< 0.001*	1.21	(0.87, 1.67)	0.260
Others	0.72	(0.34, 1.52)	0.384	2.53	(1.22, 5.25)	0.013*
Housing						
Public – 3 rooms and below	1.00	Reference		1.00	Reference	
Public – 4 rooms and above	1.22	(0.93, 1.60)	0.152	1.30	(0.97, 1.74)	0.079
Private	0.88	(0.52, 1.49)	0.637	1.50	(0.89, 2.54)	0.128
Highest education attainment						
Primary	1.00	Reference		1.00	Reference	
Secondary	1.72	(1.25, 2.37)	0.001*	2.09	(1.43, 3.06)	< 0.001*
Tertiary	2.80	(1.87, 4.20)	< 0.001*	4.11	(2.60, 6.51)	< 0.001*
Preferred source of information						
Television and/or radio	1.46	(0.99, 2.15)	0.055	1.13	(0.74, 1.72)	0.569
Printed media	1.62	(1.23, 2.13)	0.001*	1.50	(1.10, 2.03)	0.009*
Websites/ Internet	1.57	(1.16, 2.11)	0.003*	1.76	(1.28, 2.43)	0.001*
Social media	1.11	(0.83, 1.47)	0.492	0.91	(0.67, 1.23)	0.529
From friends and/or colleagues	0.96	(0.68, 1.34)	0.803	0.80	(0.55, 1.17)	0.248
From family members and/or relatives	0.58	(0.41, 0.82)	0.002*	0.99	(0.67, 1.46)	0.968

*Significant difference between variable and H7N9 or MERS awareness at *P* < 0.05

Similarly, regarding MERS, those with at least secondary education (OR = 2.09, 95% C.I 1.43–3.06) or who relied on printed media or websites/ Internet for outbreak information (OR = 1.50, 95% C.I 1.10–2.03, and OR = 1.76, 95% C.I 1.28–2.43 respectively), were significantly more likely to hear of the disease. However, unlike H7N9, the Malays and other ethnic groups (OR = 1.48, 95% C.I 1.08–2.05, and OR = 2.53, 95% C.I 1.22–5.25 respectively), were significantly more likely to hear about MERS than the Chinese population. Adults aged 22 years and above (OR = 1.76, 95% C.I 1.10–2.83) were more likely to learn about MERS compared to the respondents aged 16–21 years.

### Knowledge of H7N9/MERS

The respondent’s knowledge about scientific understanding and modes of transmission of H7N9 and MERS is reported in Tables 3 and 4 respectively. “Tables 3 and 4 about here” For H7N9 (*N* = 1389), respondents scored a mean of 9.2 (S.D ± 2.3) out of a possible maximum score of 20, while a mean of 5.9 (S.D ± 1.2) out of 10 was scored for MERS (*N* = 613). For H7N9, there were three key questions relating to the acquisition of H7N9 through poultry exposure. Out of those who were aware of H7N9, at least 60% of the respondents could answer the individual questions accurately. However, only 35% of them managed to answer all three questions correctly. In addition to the 53% of respondents who were not aware of H7N9, the total percentage of respondents who had inadequate knowledge of H7N9 was 83%. Similarly, inadequate knowledge of MERS is shown in Table 4. Out of the three questions regarding transmission modes of MERS, majority of the respondents could only provide correct answer to one question relating MERS transmission to being near a symptomatic infected person. A high percentage of respondents wrongfully thought that MERS can be transmitted through mosquito bites and exchanges of blood.

**Table 3:** Knowledge on the scientific understanding and modes of transmission of H7N9

*N* = 1389		Yes	No	Not sure
		*N (*%)	*N (*%)	*N (*%)
Scientific understanding of H7N9				
1	H7N9 can cause serious disease in an infected person, leading to death.	1180 (85.0)^b^	9 (0.6)	200 (14.4)
2	Has H7N9 caused any human deaths?	967 (69.6)^b^	47 (3.4)	375 (27.0)
3	Antiviral drugs used for seasonal influenza such as tamiflu are also effective against H7N9 and can cure the infection.	221 (15.9)^b^	344 (24.8)	824 (59.3)
4	There are vaccines that can prevent H7N9 infection in people^a^	348 (25.0)	376 (27.1)^b^	665 (47.9)
Modes of transmission of H7N9: Can H7N9 be transmitted through the following ways?				
5	Being near an infected person who is coughing and sneezing	1164 (83.8)^b^	61 (4.4)	164 (11.8)
6	Being in the same room as another person who is infected, even if he does not have symptoms yet	838 (60.3)^b^	205 (14.8)	346 (24.9)
7	Bites by mosquitos which are carrying the H7N9 virus	664 (47.8)	357 (25.7)^b^	368 (26.5)
8	Exchanges of blood (e.g. injection or transfusion)	1030 (74.2)	121 (8.7)^b^	238 (17.1)
9	Eating properly prepared and cooked chicken meat	234 (16.8)	844 (60.8)^b^	311 (22.4)
10	Touching chicken, ducks or other poultry that look ill	954 (68.7)^b^	182 (13.1)	253 (18.2)
11	Touching infected chicken, ducks or other poultry, even if they appear healthy	941 (67.8)^b^	174 (12.5)	274 (19.7)
12	Touching surfaces where the H7N9 virus is present (e.g. table tops, door handles, lift buttons)	755 (54.3)^b^	308 (22.2)	326 (23.5)

^a^At time of interview, there were no vaccines available

^b^Indicates the number and percentage of respondents who got the question correct

**Table 4:** Knowledge on the scientific understanding and modes of transmission of MERS

*N* = 613		Yes	No	Not sure
		*N (*%)	*N (*%)	*N (*%)
Scientific understanding of MERS-CoV				
1	MERS-CoV can cause serious disease in an infected person, leading to death.	539 (87.9)^b^	5 (0.8)	69 (11.3)
2	There are vaccines that can prevent MERS-CoV infection in people^a^	113 (18.4)	224 (36.6)^b^	276 (45.0)
3	Has MERS-CoV caused any human deaths?	460 (75.0)^b^	45 (4.1)	128 (20.9)
Modes of transmission of MERS-CoV: Can MERS-CoV be transmitted through the following ways?				
4	Being near an infected person who is coughing and sneezing	509 (83.0)^b^	20 (3.3)	84 (13.7)
5	Bites by mosquitos which are carrying the new Coronavirus	308 (50.2)	161 (26.3)^b^	144 (23.5)
6	Exchanges of blood (e.g. injection or transfusion)	452 (73.7)	53 (8.7)^b^	108 (17.6)

^a^At time of interview, there were no vaccines available

^b^Indicates the number and percentage of respondents who got the question correct

Frequency distribution of the respondents’ total knowledge score for H7N9 and MERS approximated a normal distribution, allowing the use of linear regression model for further analysis. After adjusting for all variables, multi-level multivariable linear regression analysis shows that among those who have heard of H7N9 or MERS, age group and ethnicity were significantly correlated to their knowledge scores of the diseases (Table 5). “Table 5 about here” In particular, older age groups were found to be positively correlated with the knowledge level. However, the Malays and Indians had a poorer understanding of both diseases as compared to the Chinese population. Additionally, respondents who were aware of H7N9 and relied on their friends and/or colleagues as their source of information were most likely to have a lower knowledge of H7N9. Respondents with at least secondary education and those residing in public housing type of 4 rooms and above were found to be positively correlated with their knowledge level of MERS.

**Table 5:** Multi-level multivariable linear regression to assess contribution of each factor to H7N9/MERS knowledge scores

	H7N9			MERS		
Variable	Coefficient	95% CI	*P*-value	Coefficient	95% CI	*P*-value
Gender						
Male	0.00	Reference		0.00	Reference	
Female	0.13	(−0.15, 0.40)	0.363	0.15	(−0.04, 0.34)	0.118
Age group						
16–21	0.00	Reference		0.00	Reference	
22–39	0.34	(− 0.21, 0.89)	0.227	0.31	(−0.12, 0.75)	0.159
40–59	0.66	(0.12, 1.19)	0.016*	0.42	(0.00, 0.85)	0.049*
≥ 60	0.77	(0.15, 1.40)	0.015*	0.28	(−0.19, 0.75)	0.237
Ethnicity						
Chinese	0.00	Reference		0.00	Reference	
Malay	−0.58	(−0.95, − 0.21)	0.002*	− 0.56	(− 0.80, − 0.32)	< 0.001*
Indian	−0.47	(− 0.84, − 0.10)	0.014*	−0.36	(− 0.61, − 0.12)	0.003*
Others	0.04	(− 0.80, 0.87)	0.933	−0.18	(− 0.69, 0.33)	0.486
Housing						
Public – 3 rooms and below	0.00	Reference		0.00	Reference	
Public – 4 rooms and above	0.33	(−0.01, 0.68)	0.057	0.41	(0.18, 0.64)	< 0.001*
Private	−0.30	(− 0.89, 0.30)	0.330	0.22	(−0.16, 0.61)	0.256
Highest education attainment						
Primary	0.00	Reference		0.00	Reference	
Secondary	0.10	(−0.37, 0.57)	0.674	0.33	(0.00, 0.67)	0.049*
Tertiary	0.51	(−0.03, 1.05)	0.062	0.54	(0.16, 0.91)	0.005*
Preferred source of information						
Television and/or radio	0.00	(−0.54, 0.54)	0.994	0.03	(−0.31, 0.36)	0.872
Printed media	0.21	(−0.15, 0.57)	0.260	0.16	(−0.08, 0.41)	0.198
Websites/ Internet	0.19	(−0.19, 0.56)	0.326	0.10	(−0.16, 0.36)	0.448
Social media	0.12	(−0.22, 0.47)	0.473	0.04	(−0.19, 0.28)	0.733
From friends and/or colleagues	−0.55	(−0.99, − 0.11)	0.015*	−0.28	(− 0.61, 0.05)	0.092
From family members and/or relatives	−0.02	(−0.48, 0.44)	0.937	−0.09	(− 0.42, 0.24)	0.608

*Significant difference between variable and H7N9 or MERS knowledge scores at *P* < 0.05

### Outbreak information source

**Fig. 1:**
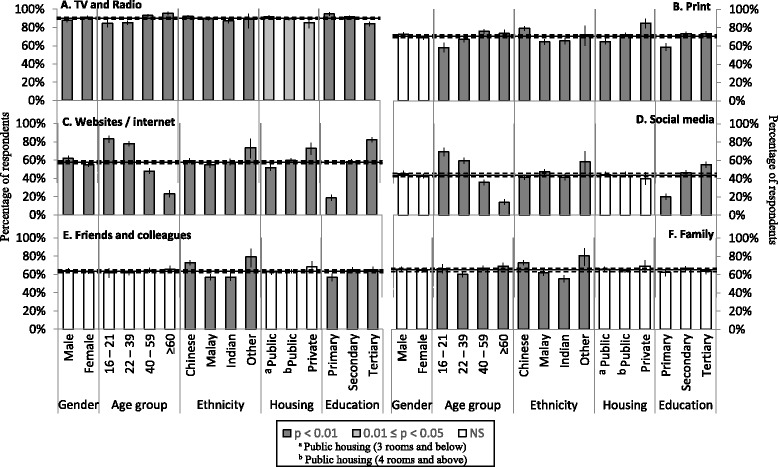
Percentage of respondents with preferred information source stratified according to socio-demographic factors

Majority of the respondents relied on traditional media channels such as television and/or radio (90.0%), and printed media (70.6%), as their information source for infectious disease outbreaks (Fig. 1). “Figure 1 about here” This is opposed to other sources such as websites/Internet (57.7%), and social media (43.9%) which were the least preferred choices. When the preferred information sources were stratified according to socio-demographic factors, the following statistically significant trends were observed: a) Age group: A higher percentage of respondents aged 40 years and above preferred television and/or radio (93.3–95.3%) and print (73.8–76.0%) as their information source as opposed to websites/ Internet (23.0–47.9%) and social media (14.1–35.8%). On the other hand, for respondents aged 16–39 years, there was a stronger preference for television and/or radio (84.5–85.2%) and websites/ Internet (78.1–83.1%) compared to print (58.3–67.3%) and social media (59.7–69.3%); b) Ethnicity: For Chinese, Malays and Indians, the top two preferred information sources were television and/or radio (92.1%, 89.7% and 87.1% respectively) and print (79.2%, 64.5% and 65.5% respectively). However, for the other minority races, television and/or radio (88.9%) and family (80.6%) were their top two choices; c) Housing: The percentages of the respondents who preferred television and/or radio were generally high (85.1–91.8%) regardless of housing type. On the other hand, there is a stronger preference for print and websites/internet as information sources among respondents staying in private housing (85.1% and 72.8% respectively) compared to public housing (64.8–72.5% and 51.7–59.6% respectively); d) Education: As the education level increased from primary to tertiary, the preference for print (58.8% to 73.5%), websites/ Internet (18.5% to 82.4%), social media (20.0% to 55.3%) and friends and colleagues (56.7% to 65.2%) as information sources increased too. However, the reverse trend was observed for television and/or radio, as 94.8% of the respondents with primary education preferred it as their information source as opposed to 84.1% of the respondents with tertiary education.

## Discussion

The results showed an apparent lack of awareness on emerging infectious agents among the respondents surveyed, with only 46.8% and 20.6% having heard of H7N9 and MERS respectively. Among those aware of H7N9 or MERS, many had multiple misconceptions, as evident from their low knowledge scores, in particular for H7N9. Of particular concern is the lack of knowledge about transmission of H7N9 via poultry exposure. Despite the fact that risk of H7N9 infection was known to be highly attributed to poultry exposure, detailed questioning revealed that less than 40% of those aware of H7N9 could correctly answer all three questions related to acquisition of the infection via poultry exposure. Likewise for MERS, a higher proportion had misunderstood that transmission could occur through blood exchanges and mosquito bites. Majority of respondents were also unsure of the use of antiviral drugs to treat H7N9 and the current lack of vaccinations to prevent both infections.

The study showed a significant association between higher education levels (at least secondary education) and awareness of H7N9 or MERS, and similarly for knowledge scores on MERS. Such trends were observed in other studies that examined the association between education level and knowledge about infectious diseases [17, 36, 37]. As education is a major social determinant of health, particularly in health promotion and disease prevention [38], educating an individual on the requisite knowledge and skills will enable them to better protect themselves and their family by avoiding high-risk health behaviors.

The results also indicated that the older age groups had higher odds of hearing about and having better understanding and knowledge of H7N9 or MERS. Further subgroup analysis in our study found that the highest percentage of respondents who chose print media as their outbreak information source were between age 40–59 (76%). This is in line with the findings by Ahlers [39] and Shah et al. [40], who reported that the older generation place greater reliance on print media. We thus postulate that, as a result of regular exposure to print media, the older age groups were more likely to receive reported news of outbreaks in the print media, contributing to their greater awareness and understanding of H7N9 or MERS. However, the effects of age persisted, even when we adjusted for effects of different media sources, probably because of residual confounding, since the questions about media sources only assessed if they did or did not rely on particular media channels but did not assess the degree of exposure, which was likely different amongst the age groups.

There were significant associations observed between ethnicity and the level of awareness and knowledge of H7N9 or MERS in Singapore. Minority ethnic groups were less likely to hear about H7N9 as compared to the Chinese majority ethnic population. Moreover, among those who have heard of H7N9, the Malays and Indians also had lower knowledge scores. This corroborates the findings of a study conducted in Malaysia, where the Malay ethnic group was found to possess a lower knowledge of H1N1 [18]. However, this differs from the findings from a Severe Acute Respiratory Syndrome (SARS) public knowledge study done in Singapore, which reported no significant associations between knowledge level and ethnicity [17]. Interestingly, the Malays and other ethnic groups were more likely to hear about MERS, but had lower knowledge scores on MERS as compared to the Chinese. Our findings could be due to a varied sense of risk perception resulting from the disparate media coverage in language mediums favoured by different ethnic groups in Singapore. Information of any new H7N9 infected cases could have received greater media coverage in the Chinese news given that cases occurred mainly in China. Moreover, minority ethnic groups were less likely to travel to China, and thus may not be as motivated to learn more about H7N9. Likewise for MERS, infections have been mainly located in Saudi Arabia and the Middle East [41] and hence, there was a specific health advisory, released by the Ministry of Health (MOH) in Singapore, targeting Umrah and Haj pilgrims traveling to Islamic pilgrimage sites in the Arabian Peninsula [42]. This could have caused a heightened awareness of MERS among the Malay Muslim community.

In terms of outbreak information source, the television and radio were found to be the most utilized among the respondents regardless of socio-demographic factors. Recent studies on outbreak information dissemination had reported traditional media channels to still be the main source of information, with minimal health information exchanged on social media [43–46]. Our findings are also congruent with the study conducted by Vijaya et al. [17] where the authors found that majority of the respondents depended on the television and printed media for timely and accurate information during the SARS outbreak in Singapore. Based on our results, print, websites/ Internet and social media could act as complementary information sources in Singapore but they should be targeted towards specific socio-demographic groups. For individuals aged 40 years and above or staying in private housing, print media were also preferred in addition to television and radio. Websites/ Internet were well utilized by individuals aged 16–39 years or with a tertiary education, while the use of social media as an outbreak information source was mainly observed in individuals aged 16–21 years old. In addition, respondents were most likely to have lower knowledge scores on H7N9 if they chose to hear from their friends and colleagues. This is substantiated by Scanfeld et al. [47] who reported that the public can be misled by inaccurate health information disseminated through word of mouth and social media.

As highlighted in the previous paragraphs, demographics contribute to significant differences in awareness and knowledge regarding emerging infections. These differences suggest a need to consider audience segmentation in the design and dissemination phase in order to convey outbreak information more effectively. Having a limited portion of people being aware and well-informed of these viruses in times of outbreak might result in panic and misleading information being disseminated through social channels. This could lead to adoption of unwanted practices instead of accurate preventive measures. Interestingly, during the outbreak of SARS, knowledge level on the disease itself was not associated with adoption of preventive measure but public trust was. Such studies [48, 49] highlight the fact that it is essential in the event of an outbreak, where responses need to be swift, for the public to be able to access accurate information on preventive measures through reliable channels in order to respond accordingly. Finally, identification and training of community leaders and motivated individuals on outbreak preparedness might further complement traditional media as another source of information.

There are strengths and limitations to the study. As respondents were recruited from diabetic and multi-ethnic cohorts, it has allowed us to assess our study objectives in a more vulnerable population group and also identify differences between ethnicities and note the need to consider audience segmentation during future outbreak communication. On the other hand, during the process of data collection, sources of bias include potential selection bias of respondents, as respondents were asked if they were willing to participate in the survey, resulting in volunteer bias and may not be truly representative of the general Singapore population. In addition, convenience sampling of respondents who were household members of contacted members of the original cohorts that our sampling strategy drew on comprised 78% of the total data set, resulting in grouped participants in the same household. This was partially accounted for by using the multilevel mixed-effects model with a random intercept term. Finally, the survey was conducted between June to December 2013, a period shortly after the news of the first case of human H7N9 was reported in March 2013 but almost a year after the first case of MERS was reported in September 2012. This temporal difference could have varied the duration of risk communication and public engagement for both infections at the point of survey and hence, impacted the level of awareness and knowledge on H7N9 or MERS.

## Conclusion

As a multicultural society, Singapore presents a unique set of challenges for successful risk communication. Results of this study suggest that public health communication and risk dissemination regarding H7N9 or MERS were not optimal in Singapore. Public health education about infectious disease outbreaks should reach out more to the younger population, lower educated groups and ethnic minorities to equip them with better information on specific preventive measures. Despite the growing popularity of social media in Singapore, traditional media channels such as the television, radio, printed media as well as websites remain the primary source of outbreak information among respondents in this study. Future health communication strategies for emerging infectious diseases should consider audience segmentation and the most suitable media channels for disseminating risk information across various socio-demographic groups.

Moving forward, the study proposes that a concerted effort be orchestrated between the media and health authorities, to seamlessly communicate information through news articles about specific preventive measures the public can take to protect themselves during outbreaks. Future surveys should try to understand how local communities network and communicate and the barriers and facilitators for individuals to take action in the event of an outbreak. More studies should also be done to analyse the effectiveness of tailored messages. Qualitative studies in the form of focus groups may be useful to pre-test and assess the responses of multi-cultural audiences regarding uptake of information. This will benefit the design of health communications during early stages of development. Additionally, in-depth interviews can also be employed to elicit personal responses and concerns of the messages, for individuals who may be difficult to reach, particularly vulnerable groups who have limited writing and reading skills.

## Abbreviations

MERS: Middle East respiratory syndrome
OR: Odds ratios
SARS: Severe acute respiratory syndrome
SSHSPH: Saw swee hock school of public health
